# MLL is required for miRNA-mediated translational repression

**DOI:** 10.1038/s41421-019-0111-0

**Published:** 2019-09-03

**Authors:** Shouhai Zhu, Zhihong Chen, Ruiheng Wang, Yuting Tan, Maolin Ge, Yan Sun, Dan Li, Yutian Hu, Chunjun Zhao, Zhu Chen, Saijuan Chen, Han Liu

**Affiliations:** State Key Laboratory of Medical Genomics, Shanghai Institute of Hematology, Rui Jin Hospital, School of Medicine and School of Life Sciences and Biotechnology, Shanghai Jiao Tong University, 200025 Shanghai, China

**Keywords:** Organelles, miRNAs, Epigenetics

Dear Editor,

The mixed-lineage leukemia (MLL) protein was originally identified through its association with acute lymphoid and myeloid leukemias^1^. MLL is an H3K4 histone methyltransferase that can execute methylation through its evolutionarily conserved SET domain, and this activity is essential for normal MLL function^2^. MLL is proteolytically cleaved into two fragments: MLL^N320^ and MLL^C180^, which non-covalently interact to form an intramolecular complex^3^.

MLL is regarded as a nuclear protein and several nuclear localization signals in MLL^N320^ contribute to its nuclear localization;^4^ therefore, nuclear extracts are routinely used to purify MLL protein complexes^2,5^. Through this strategy, a number of nuclear proteins such as MENIN, ASH2L, and WDR5 have been identified as essential MLL-binding partners^5,6^. However, previous studies showed that the cleavage of MLL by Taspase1 occurred in the cytoplasm^3^ and that free MLL^C180^ could be exported to the cytoplasm^7^, thus raising the possibility that MLL might possess some underappreciated functions in the cytoplasm. To identify possible MLL-binding proteins in a more comprehensive manner, total 293T cell extracts were used for MLL affinity purification followed by LC/MS analysis. As expected, we acquired a number of previously identified MLL-binding proteins including MENIN, ASH2L, and WDR5^6^. Interesting, we repeatedly identified several cytoplasmic proteins, including P-body components such as YB-1, EDC3, DDX6, UPF1, and PABPC1 (Supplementary Table S1), suggesting a physical interaction between MLL and these P-body components.

In mammalian cells, P-bodies contain nontranslated mRNAs and the conserved core of proteins involved in the decay and translational repression of mRNAs^8^. These factors include the decapping enzymes DCP1A and DCP2, the decapping activators EDC3, EDC4, and DDX6^8^. To verify the connections between MLL and P-bodies, we examined several P-body marker proteins including DCP1A, DDX6, EDC3, and EDC4 in endogenous MLL immunoprecipitates, and confirmed a physical interaction between MLL and these P-body components (Fig. 1a and Supplementary Fig. S1a). Furthermore, we demonstrated that these interactions preferentially occur in cytoplasm, but not in nucleus (Supplementary Fig. S1b).

**Fig. 1:**
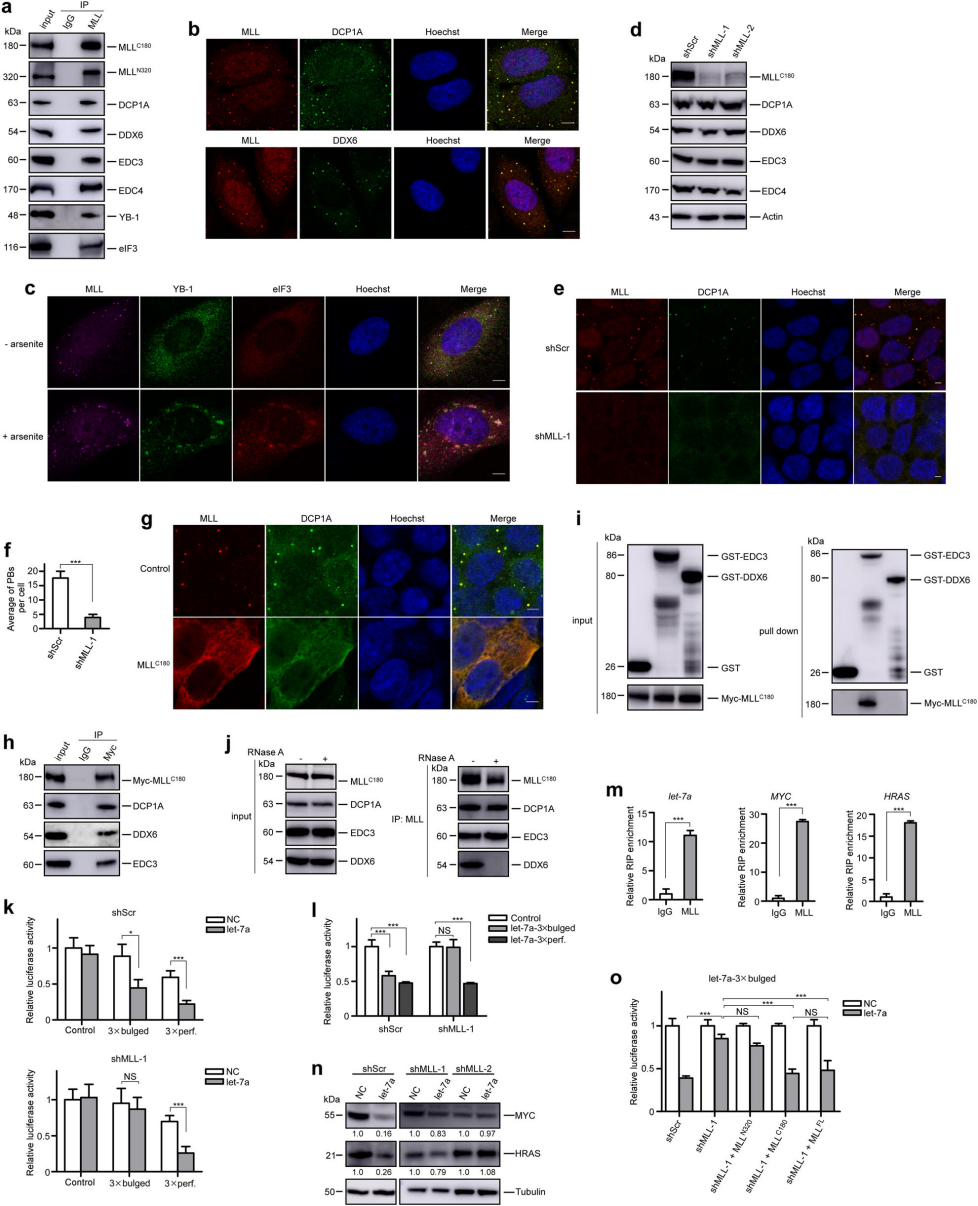
MLL localizes to P-bodies and is essential for P-body integrity. **a** The total lysates of 293T cells were prepared and subjected to immunoprecipitation using anti-MLL antibodies. Copurified proteins were examined by immunoblots using the indicated antibodies. Specific antibodies were used to detect the MLL^N320^ and MLL^C180^, respectively. **b** The localization of endogenous MLL and P-body markers in 293T cells was visualized by immunofluorescence using indicated antibodies. Scale bar, 5 μm. **c** 293T cells were untreated (upper panels) or arsenite treated (0.5 mM, 45 min) (lower panels), then fixed and stained with indicated antibodies. Note that YB-1 is detectable in P-bodies in unstressed cells, and relocalized to stress granule upon arsenite treatment, whereas eIF3 is specific for stress granule. **d** Knockdown of MLL by targeting three independent sequences and its effect on the expression of P-body proteins were examined by western blot using indicated antibodies. **e** 293T-shScr and 293T-shMLL cells were examined by immunofluorescence using antibodies against MLL and DCP1A. Scale bar, 5 μm. **f** Detectable P-bodies detected by immunofluorescence using antibodies against DCP1A were quantified for all cells in the field of view (×126 magnification) and data from at least three random fields were collected and analyzed using Image J. **g** MLL^C180^ alone was overexpressed in 293T cells, then immunofluorescence experiments were performed using antibodies against MLL^C180^ (Bethyl) and DCP1A. Scale bar, 5 μm. **h** 293T cells were transfected with *MLL*^*C180*^, cell cytoplasm fractions were prepared for the coimmunoprecipitation assays using the indicated antibodies. **i** Direct interactions between MLL^C180^ and GST-EDC3, GST-DDX6 were examined. Left panels: western blots shown the inputs of purified GST-EDC3, GST-DDX6, and Myc-MLL^C180^. Right panels: the pull down immunoblots were shown with GST-EDC3 and GST-DDX6 as the baits and the pulled MLL^C180^ detected by an anti-Myc antibody. **j** 293T cell lysates were treated with RNase A followed by anti-MLL immunoprecipitation. Western blots were performed using indicated antibodies. **k** 293T-shScr and shMLL cells transfected with Agomir-negative control (NC) or Agomir-let-7a mimic (let-7a) were subjected to dual luciferase reporter assays. The ratio of luciferase activity was measured and normalized to the value of the cells transfected with the control reporter and NC. **l** The effects of *MLL* knockdown on the function of endogenous *let-7a* were analyzed using dual luciferase reporter assays. The ratio of luciferase activity was measured and normalized to the value of the cells transfected with the control reporter in each cell line. **m** Lysates of 293T cells were prepared and subjected to anti-MLL RIP or mock RIP using IgG. Pull-downed RNAs were analyzed by qRT-PCR. **n** 293T-shScr and 293T-shMLL cells were transfected with NC and let-7a. After 24 h of transfection, cell lysates were prepared and assessed by anti-MYC and anti-HRAS western blots. The ratio of protein levels was normalized to the value of the cells transfected with the NC. **o** 293T-shMLL cells were transfected with shRNA-resistant *MLL*^*C180*^, *MLL*^*N320*^ or full-length *MLL* (MLL^FL^) to determine which of these subunits could relief the dysregulated *let-7a* mediated gene silencing due to *MLL* knockdown. Experiments were performed as described in Fig. 1k. The ratio of luciferase activity was measured and normalized to the value of the cells transfected with the NC. *P < 0.05, **P < 0.01, ***P < 0.001. NS, no significant difference. Data represent mean and s.e.m of three independent experiments

To determine whether MLL localizes to P-body foci, we examined the localization of MLL. Although MLL dominantly localized to the nucleus, a significant portion of MLL was present in discrete cytoplasmic foci, colocalizing with P-body marker proteins such as DCP1A, DDX6, EDC3, and EDC4 (Fig. 1b and Supplementary Figs. S1c–e). P-bodies and stress granules (SGs) are two closely related cytoplasmic mRNP granules, sharing some common components such as YB-1^9^. YB-1, which partially presented in P-bodies when cells were untreated, predominantly relocalized into SGs after sodium arsenite treatment^10^. Since our results showed that YB-1 as well as SG marker eIF3 was binding partner of MLL (Fig. 1a and Supplementary Fig. S1a), we also investigated whether MLL was a component of SGs. We found that MLL colocalized with a portion of YB-1 in untreated cells. Upon sodium arsenite treatment, part of MLL together with the majority of YB-1 relocalized to SGs as indicated by the SG marker eIF3, while the rest of the cytoplasmic MLL mainly remained in the P-bodies with DCP1A (Fig. 1c and Supplementary Fig. S1f). Thus, these results demonstrated that MLL was present not only in P-bodies but also in the closely related SGs.

Previous studies have shown that different components of P-bodies have various effects on P-body integrity^9^. We sought to determine the role of MLL on P-body integrity by examining the effects of MLL depletion on the formation of P-bodies in *MLL* knockdown 293T cells and *Mll* knockout MEF cells. Depletion of MLL caused a significant decrease in DCP1A- or DDX6-associated P-bodies without affecting the protein levels of P-body components (Fig. 1d–f and Supplementary Fig. S1g–l). These results revealed that MLL was required for the maintenance of P-bodies.

We next investigated which MLL subunit was involved in P-body assembly. Consistent with previous reports, ectopically expressed MLL^N320^ and MLL^C180^ alone were predominantly localized in the nucleus and the cytoplasm, respectively (Fig. 1g and Supplementary Fig. S2a, b). In contrast, MLL^C180^ from ectopically expressed full-length MLL was predominantly localized to the nucleus (Supplementary Fig. S2c, d), suggesting that MLL^C180^ was normally constrained in the nucleus by MLL^N320^. Interestingly, overexpression of MLL^C180^ alone led to disruption of microscopic P-body foci without affecting the protein levels of P-body components; however, MLL^C180^ still remained colocalized with P-body components in a diffuse pattern (Fig. 1g, h and Supplementary Fig. S2b, e). We further demonstrated that EDC3, but not DDX6, could pull down MLL^C180^, indicating a direct interaction between MLL^C180^ and at least some of the P-body components (Fig. 1i). Co-IP experiments further revealed that the interaction between MLL and DDX6 decreased dramatically after RNase A treatment, indicating that this interaction was an RNA-dependent indirect interaction, rather than a direct protein–protein interaction (Fig. 1j and Supplementary Fig. S2f). In contrast, the interactions between MLL and other P-body components including DCP1A and EDC3 were not affected by RNase A treatment (Fig. 1j and Supplementary Fig. S2f). These results thus revealed that it was MLL^C180^ that interacted with P-body components directly and was crucial for maintaining P-body integrity.

miRNA-mediated gene silencing takes place in the P-bodies^11^. However, the loss of visible P-body foci does not necessarily mean a defect in miRNA-mediated gene silencing^12^. Given that depletion of MLL could affect the integrity of P-bodies, we sought to determine whether MLL depletion would affect miRNA-mediated gene silencing. Mature miRNAs cause gene silencing either by cleaving perfectly matched mRNAs or by suppressing the translation of partially matched mRNAs^13^. To determine which type of miRNA-mediated gene silencing would be affected by MLL, we used different luciferase reporters harboring perfect or bulged *let-7a* target sites to distinguish between these two types of miRNA-mediated gene silencing (Supplementary Fig. S3a). Interestingly, depletion of MLL impaired the function of *let-7a* mimics as well as endogenous *let-7a* to silence the imperfect target but had little effect on the perfect target (Fig. 1k, l and Supplementary Fig. S3b, c). The mature expression levels of the shRNA control and the MLL-targeting shRNA were not only equally abundant, but were also similarly bound to AGO2 (Supplementary Fig. S3d), thus excluding the possibility that differing reporter activity levels were caused by a competitive effect of mature shRNAs on Argonaute proteins that affected endogenous miRNAs^14^. The preferential effect of MLL in miRNA-mediated silencing of imperfect targets was further documented by *CXCR4* reporter assays (Supplementary Fig. S3a, e).

To explore which miRNAs and mRNA targets could be affected by MLL, we first performed RNA immunoprecipitation (RIP) to identify the miRNAs and their mRNA targets associated with MLL. The RIP-seq results demonstrated a plethora of MLL-binding miRNAs including *let-7a*, *miR-10a*, and *miR-196b* (Supplementary Table S2), which were further validated by qRT-PCR (Fig. 1m and Supplementary Fig. S3f). In addition, among MLL-binding mRNAs we identified *MYC* and *HRAS*, two well-characterized targets of *let-7a*^15^ (Fig. 1m and Supplementary Table S3), suggesting that translational regulation of *MYC* and *HRAS* by *let-7a* might be affected by MLL. We therefore evaluated whether the expression of MYC and HRAS proteins could escape translational suppression by *let-7a* in MLL-depleted cells. In the control cells, MYC and HRAS protein levels decreased significantly upon treatment with *let-7a* mimics. In contrast, MYC and HRAS protein expression in MLL-depleted cells showed no obvious decrease when cells were transfected with *let-7a* (Fig. 1n and Supplementary Fig. S3g). In addition, the ratio of MYC protein to mRNA was much higher in MLL-depleted cells (Supplementary Fig. S3h), indicating that translational suppression of *MYC* mRNA occurred in an MLL-dependent manner. Together with the previous reporter assays, these results suggested that MLL was required for the translational repression mediated by a subset of miRNAs, especially *let-7a*.

To demonstrate that MLL plays a causal role in the recruitment of miRNA to miRISC, we reintroduced shRNA-resistant *MLL*^*C180*^, *MLL*^*N320*^ or full-length *MLL* into *MLL* knockdown 293T cells and *Mll* knockout MEF cells and found that the introduction of MLL^C180^ but not MLL^N320^ could partially reverse the deficits in miRNA activity caused by loss of endogenous *MLL*, as evaluated by reporter assays (Fig. 1o and Supplementary Fig. S3i, j). Taken together, these results revealed that MLL plays a causal role in targeting miRNAs to form a functional miRISC complex.

A consensus has developed that mature MLL serves as an epigenetic regulator in the form of an intramolecular complex. However, our findings have revealed, for the first time to the best of our knowledge, that MLL subunits do not have to bind each other to be functional; instead, they can be separated and exert additional functions. Since MLL subunits can only be separated when MLL is processed by Taspase1, our findings reinforce the concept that the processing of MLL is vital to proper MLL function.

## Supplementary information

Supplementary Information

Supplementary Table S1

Supplementary Table S2

Supplementary Table S3

## Supplementary information

**Supplementary Information** accompanies the paper at (10.1038/s41421-019-0111-0).
