# Supplementary material for: MLL is required for miRNA-mediated translational repression
Source: Cell Discov. 2019 Sep 3;5:43. doi: 10.1038/s41421-019-0111-0 (PMC6796902; doi:10.1038/s41421-019-0111-0)
Supplement: Supplementary file 1 — Supplementary Information [file 41421_2019_111_MOESM1_ESM.pdf]

Supplementary Information

Materials and methods

Construction of plasmids. To generate pmirGLO dual luciferase reporters whose expression is controlled by let-7a, we inserted annealed primers into the *Sac*I-*Xba*I sites of pmirGLO miRNA target expression vector (Promega) to get constructs having three perfectly base pairing (CAACTATACAACCTACTACCTCAAACTATACAACCTACTACCTCAAACTA TACAACCTACTACCTCAT; pmirGLO-3xperfect) and three bulged (CGCACAGCCTATTGAACTACCTCACTCGGAGCACAGCCTATTGAACTAC CTCAGGCCTGCACAGCCTATTGAACTACCTCAT: pmirGLO-3xbulged) let-7a sites in the 3'UTR region. To construct shRNA vectors for *MLL*, hairpin-encoding oligonucleotides against *MLL* (GTGCCAAGCACTGTCGAAA; GCCAAGCACTGTCGAAATTAC) were annealed and ligated into pLKO.1 vector. Vectors expressing full-length MLL, Taspase1 cleavage sites mutated MLL, MLL^{N320} or MLL^{C180} has been described previously[1, 2]. shRNA-resistant vectors were created by site-directed mutagenesis. GST fusion constructs were generated by amplifying the *EDC3* or *DDX6* sequence into the pGEX-4T-1 (GE healthcare). The primer sets were:
EDC3:
sense: ATGGCTACAGATTGGCTGGGAAG;
antisense: CTAAGCAGAGTGCAGTGGGATAAC;
DDX6:
sense: ATGAGCACGGCCAGAACAG;
antisense: TTAAGGTTTCTCATCTTCTACAGG;

Cell culture and transfection. HEK293T, 293T-shScr and 293T-shMLL stable cell lines were cultured in Iscove's Modified Dulbecco's Medium (IMDM) supplemented with 10% fetal bovine serum and 1% 100×Pen Strep. All cell culture reagents were obtained from Life technologies. The *Mll* wild-type ($Mll^{+/+}$) and *Mll* knockout ($Mll^{-/-}$) mouse embryonic fibroblasts (MEF) cell lines were maintained as described previously[3].

For the let-7a experiments, 1 ug of let-7a mimic were used for the transfection. After 24 hours, the transfection efficiencies were validated by Western blot analysis. For plasmids transfection, FuGENE HD Transfection Reagent (Promega) was used according to the protocol.

Antibodies and Western blot. The antibodies used in this study were: anti-MYC (Santa Cruz; sc-40), anti-DCP1A (for Western blot: Sigma, D5444; for IF: Sigma, WH0055802M6), anti-DDX6 (for Western blot: GeneTex, GTX102795; for IF: Santa Cruz, sc-376433), anti-EDC3 (for Western blot: Santa Cruz, sc-365024; for IF: Santa Cruz, sc-271806), anti-EDC4 (Santa Cruz, sc-137444),

anti-MLL (Bethyl, A300-374A, recognizing aa2725-2775 of MLL), anti-MLL1 (Cell Signaling, 14689, recognizing amino-terminal of MLL), anti-MLL1 (Sigma, PLA0100, recognizing aa2725-2775 of MLL), anti-YB-1 (for Western blot: ABclonal, A7704; for IF: Santa Cruz, sc-101198), anti-eIF3 (Santa Cruz, sc-16377), anti-RAS (BD Biosciences, 610001), anti-Actin (Sigma, A1978) , anti-Tubulin (Sigma, T0198), anti-GAPDH (Sigma, G8795) and MLL-CT antibody that recognizes MLL^{C180} (aa2829-2883) has been described previously[1]. Unless specified, MLL-CT was used for Western blot, IP or IF. For co-IP assay, secondary antibodies used for immunoblot were Rabbit or Mouse TrueBlot antibodies (Rockland) to eliminate interference by the heavy and light chains of the immunoprecipitated antibody. All the antibodies were used according to the manufacturer's recommendations. Antibodies were detected using the enhanced chemiluminescence method (Western Lightning, PerkinElmer) and immunoblot signals were acquired with the AI600 Imaging system (GE).

Immunoprecipitation (IP) and LC-MS/MS analysis. 293T cells at 80% confluency were harvested, washed once in PBS and lysed in Cell Lysis Buffer (CST) or RIPA lysis buffer (CST) with freshly added protease inhibitor cocktail (Roche) for 10 min at 4℃. The lysates were briefly sonicated and clarified by centrifugation at 12,000rpm for 10min. The supernatant were incubated with Dynabeads protein G beads (Life technologies) and indicated antibodies at 4℃ for overnight with rotation. Beads were washed 4 times in lysis buffer and bound proteins eluted by the addition of 1× LDS sample buffer (Life technologies). The eluates were analyzed by immunoblot. To examine the RNA dependence of protein-protein interactions, lysates were treated with 0.2 ug/ul RNase A for 20 min at room temperature before immunoprecipitation. Nuclear-cytoplasmic fractionation was performed using the NE-PER kit (Thermo Fisher).

For LC-MS/MS (Liquid Chromatography-Mass Spectrometry/Mass Spectrometry) analysis, 293T cells were transfected with full length MLL expressing plasmids. After 24 hours, these cells were lysed with RIPA buffer (CST) with freshly added protease inhibitor cocktail (Roche), and then proteins were immunoprecipitated with anti-MLL antibody. Immunoprecipitated proteins were digested with trypsin (Promega) and analyzed by ultimate capillary LC system (Dionex) coupled with LTO Orbitrap mass analyzer (Thermo Fisher). The results were searched on MassMatrix against human UniProt database.

Immunofluorescence (IF). For immunofluorescence, cells were fixed with 4% paraformaldehyde in PBS for 30 min at room temperature and permeabilized in 0.2% (v/v) Triton X-100 in PBS for 20 min at room temperature. The nonspecific binding was blocked for 60 min in PBS containing 1% BSA and 0.1% Tween-20 and probed in the same buffer with primary antibodies overnight at 4 ℃. The slides were washed 3 times in PBS containing 0.1% Tween-20 and were incubated with the Alexa-labelled secondary antibodies (Thermo Fisher) and

Hoechst33342 (Thermo Fisher) for 1 hour. The coverslips were mounted in Fluoromount Aqueous Mounting Medium (Sigma) and imaged using TCS SP8 confocal microscopes (Leica). For z-stack analysis, optical sections were obtained along with the z axis at 0.5 um intervals. Images were analyzed using the Image J software.

RNA isolation and qRT-PCR. miRNA was isolated using *mir*Vana miRNA Isolation Kit (Life Technologies) and dissolved in the appropriate amount of RNase-free water as indicated. mRNA was extracted according to the manufacturer's instructions (Sangon). RNAs concentration was measured by QuantiFluor RNA System (Promega). Reverse transcription was performed using GoScript Reverse Transcription System (Promega). qPCR was performed with Power SYBR Green master mix (Applied Biosystems) in triplicate using the following primer sets:

hsa-let-7a-5p

sense: AAGGCGGTGAGGTAGTAGGTTGT;

antisense: ATCCAGTGCAGGGTCCGAGG;

reverse transcription primer:

GTCGTATCCAGTGCAGGGTCCGAGGTATTCGCACTGGATACGACAACTAT.

hsa-miR-10a-5p

sense: GCGCGTACCCTGTAGATCCGAA;

antisense: ATCCAGTGCAGGGTCCGAGG;

reverse transcription primer:

GTCGTATCCAGTGCAGGGTCCGAGGTATTCGCACTGGATACGACCACAAA.

hsa-miR-196b-5p

sense: GCGCGCGTAGGTAGTTTCCTGTT;

antisense: ATCCAGTGCAGGGTCCGAGG;

reverse transcription primer:

GTCGTATCCAGTGCAGGGTCCGAGGTATTCGCACTGGATACGACCCCAAC.

MYC

sense: AATGAAAAGGCCCCCAAGGTAGTTATCC;

antisense: GTCGTTTCCGCAACAAGTCCTCTTC.

HRAS

sense: ATGACGGAATATAAGCTGGTGGT;

antisense: GGCACGTCTCCCCATCAATG.

GAPDH

sense: CATGTTCGTCATGGGGTGAACCA;

antisense: AGTGATGGCATGGACTGTGGTCAT.

Luciferase reporter assay. Cells were transfected with dual luciferase reporters containing three matched or mismatched *let-7a* binding sites and Agomir-negative control (NC) or Agomir-let-7a-5p mimic (let-7a). At 24 h post-

transfection, cells were harvested and dual luciferase reporter assays (Promega) were performed according to manufacturer's protocol and quantified with a GloMax 20/20 Luminometer (Promega). The *CXCR4* reporter assays were performed as described previously[4].

RIP and RIP-seq. For RNA immunoprecipitation (RIP) experiment, about 10 million cells were collected, pelleted by centrifuge for 5 min at 1,000 g and washed once with cold PBS (5 ml). The cell pellets were re-suspended with 500 ul lysis buffer (140 mM NaCl, 50 mM Tris-HCl pH 8.0, 0.5% Triton X-100) with protease inhibitor cocktail (Roche) and 400U/ml RNase inhibitor (Promega). Then the mRNP lysate was incubated on ice for 10 min and centrifuged at 12,000 rpm for 10 min. Cell lysate was incubated with indicated antibodies and Dynabeads protein G (Life technologies); the tube was rotated continuously at 4 ℃ overnight. The beads were collected, washed 4 times with 1 ml ice-cold lysis buffer with RNase inhibitor. Then total RNAs were isolated for qRT-PCR or RNA-sequencing. For RIP-seq, isolated RNAs were sequenced using HiSeq2000 (Illumina). The sequence reads were aligned to the Ensembl human genome, then the aligned read counts were quantified and normalized. The RIP-seq raw data have been deposited in the Gene Expression Omnibus. All the data are accessible under GSE115848 (GSM3191828 is the total RNA sample and GSM3191829 is the small RNA sample).

GST Pull-down Assay. For the GST pull-down assay, Glutathione Agarose (Thermo Scientific) slurry was saturated with purified GST-EDC3 or GST-DDX6 at 4°C overnight. 293T cells were transfected with Myc-MLL^{C180}, 24 hours after transfection, cell lysates were collected then incubated with GST proteins at 4°C for 4 hours. The bound proteins were eluted with 1× LDS sample buffer and then analyzed by Western blot.

Lentivirus Production and Infection. Lentivirus for sh*MLL* as well as their controls were packaged with pMD2.G and pSPAX2. Briefly, 3 μg pMD2.G, 9 μg pSPAX2 and 12 μg lentivirus plasmids were co-transfected into 293T cells in 10 cm cell culture dish using ProFection Mammalian Transfection System (Promega). The lentivirus particles were harvested at 48 hours after transfection and concentrated. For infection, the concentrated lentivirus particles were added into the medium with 8 ug/ml polybrene and cultured at 37°C for 24-48 hours before they were washed out.

Statistical analysis. Data are displayed as the mean ± s.e.m., and *P*-values were calculated using two-tailed Student *t*-tests. The statistically significance level is indicated as * for $P<0.05$, ** for $P<0.01$, or *** for $P<0.001$.

Supplementary Reference:

1. Liu, H., Cheng, E. H. & Hsieh, J. J. Bimodal degradation of MLL by SCF^{Skp2} and APC^{Cdc20} assures cell cycle execution: a critical regulatory circuit lost in leukemogenic MLL fusions. *Genes Dev.* **21**, 2385-2398 (2007).
2. Hsieh, J. J., Cheng, E. H. & Korsmeyer, S. J. Taspase1: a threonine aspartase required for cleavage of MLL and proper HOX gene expression. *Cell* **115**, 293-303 (2003).
3. Liu, H. *et al.* Phosphorylation of MLL by ATR is required for execution of mammalian S-phase checkpoint. *Nature* **467**, 343-346 (2010).
4. Doench, J. G., Petersen, C. P. & Sharp, P. A. siRNAs can function as miRNAs. *Genes Dev.* **17**, 438-442 (2003).

Supplementary Figures and Figure Legends

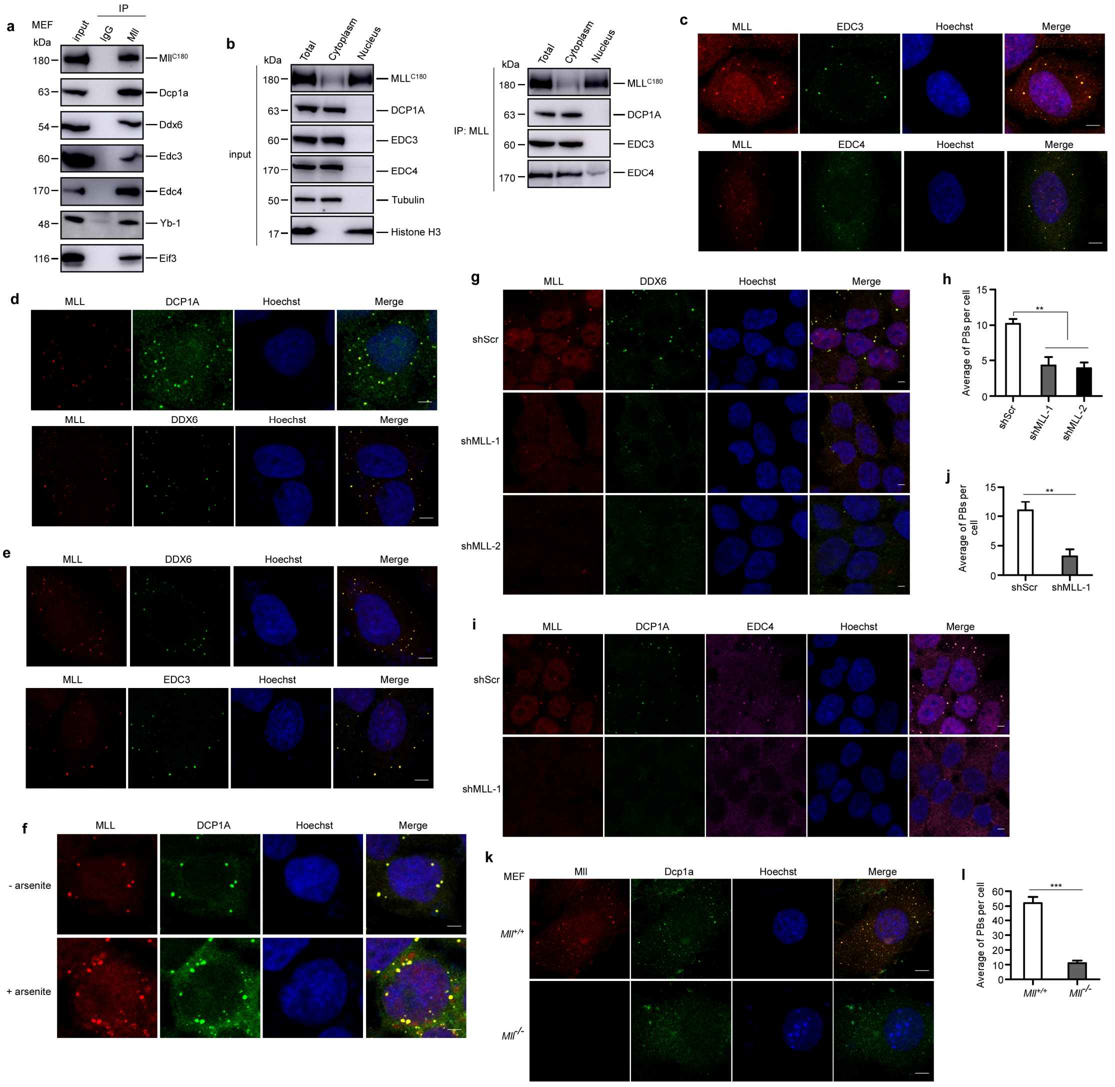

Supplementary Fig. S1 MLL localizes to P-bodies and is essential for P-body integrity. a Total lysate of MEF cells were prepared and subjected to immunoprecipitation using anti-Mll antibodies. Co-purified proteins were examined by immunoblots using the indicated antibodies. **b** The cytosolic and nuclear fractions of 293T cells were separated and subjected to immunoprecipitation using anti-MLL antibodies. Co-purified proteins were examined by immunoblots using the indicated antibodies. **c-e** 293T cells were probed for endogenous MLL using three different anti-MLL antibodies: MLL-CT (**c**), MLL (Bethyl) (**d**) and MLL1 (Sigma) (**e**). Anti-DCP1A, DDX6, EDC3, EDC4 antibodies were used as P-bodies markers. Hoechst33324 (blue) was used to mark the nucleus. Scale bar, 5 um. **f** 293T cells were untreated or treated with 0.5 mM arsenite for 45 min, then immunofluorescence experiments were performed using anti-MLL and anti-DCP1A antibodies. Scale bar, 5 um. **g-h** 293T-shScr and 293T-shMLL cells were examined by immunofluorescence using antibodies against MLL (Bethyl) and DDX6 (**g**). Scale bar, 5 um.

Detectable P-bodies detected by immunofluorescence were quantified for all cells in the field of view (×126 magnification) and data from at least 3 random fields were collected and analyzed using Image J (**h**). **i-j** 293T-shScr and 293T-shMLL cells were examined by immunofluorescence using antibodies against MLL (Bethyl) and DCP1A, EDC4 (**i**). Scale bar, 5 um. Detectable P-bodies detected by immunofluorescence were quantified for all cells in the field of view (×126 magnification) and data from at least 3 random fields were collected and analyzed using Image J (**j**). **k-l** *Mll* wild-type ($Mll^{+/+}$) and *Mll* knockout ($Mll^{-/-}$) MEF cells were examined by immunofluorescence using antibodies against MLL (Bethyl) and DCP1A (**k**). Scale bar, 5 um. Detectable P-bodies detected by immunofluorescence were quantified for all cells in the field of view (×126 magnification) and data from at least 3 random fields were collected and analyzed using Image J (**l**). ** for $P<0.01$, *** for $P<0.001$. Mean and s.e.m.

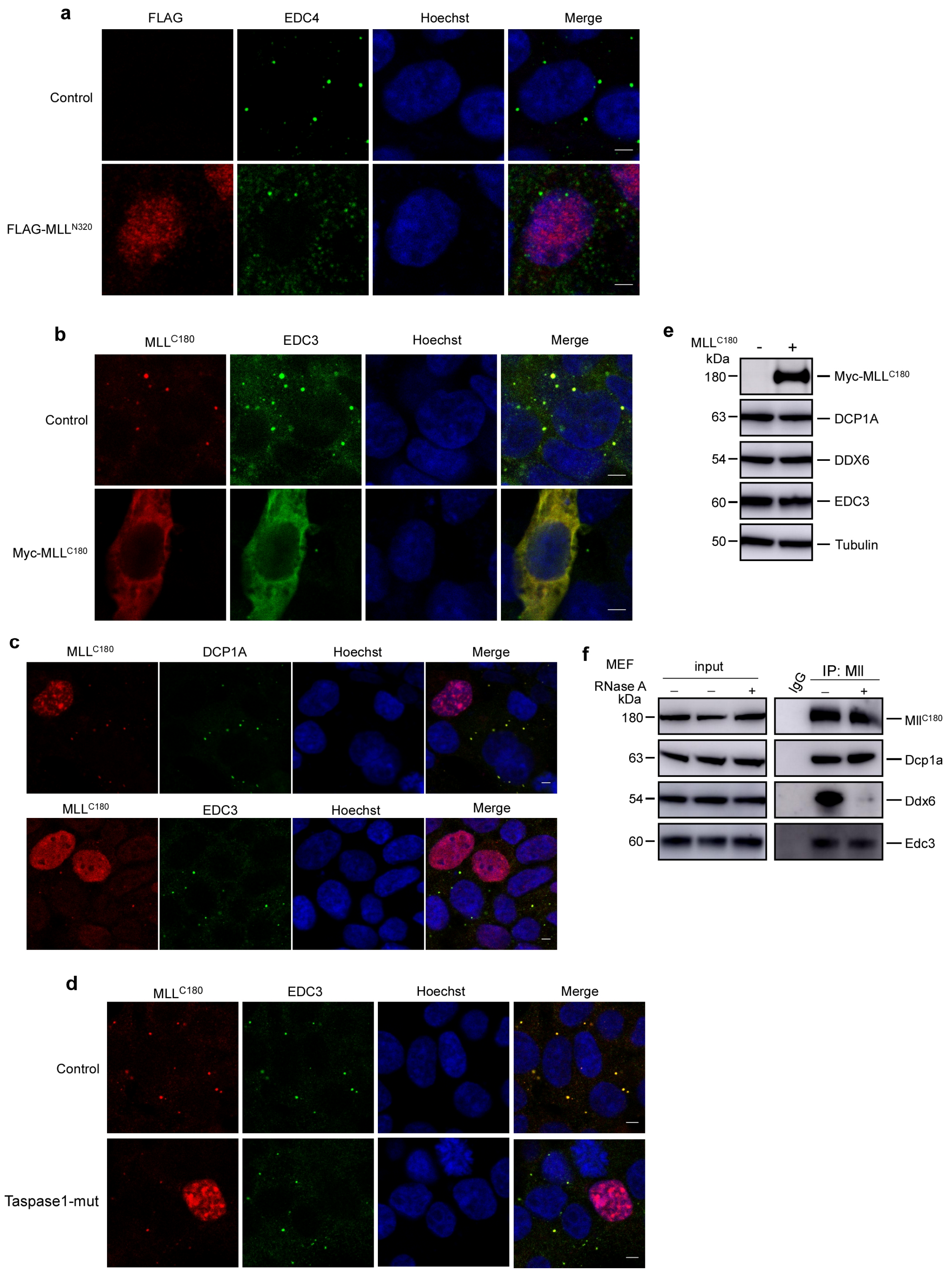

Supplementary Fig. S2 The cellular localization of MLL^{N320} and MLL^{C180}. a-b FLAG-MLL^{N320} and Myc-MLL^{C180} were ectopic expressed in 293T cells. The localization of MLL^{N320} and MLL^{C180} were examined by immunofluorescence using antibodies against FLAG tag and MLL^{C180}, respectively. Scale bar, 5 um. **c** 293T cells transfected with full length MLL were examined by immunofluorescence with antibodies against MLL^{C180}, DCP1A and EDC3,

respectively. Scale bar, 5 um. **d** 293T cells were transfected with the Taspase1 cleavage sites mutated MLL expressing vector. The localization of full length MLL were examined by immunofluorescence with antibodies against MLL^{C180} and EDC3. Scale bar, 5 um. **e** The expression of Myc tagged MLL^{C180} and P-bodies marker proteins were evaluated by Western blot. **f** MEF cell lysates were untreated or treated with RNase A followed by anti-Mll immunoprecipitation. Immunopurified complexes were analyzed by Western blot with indicated antibodies.

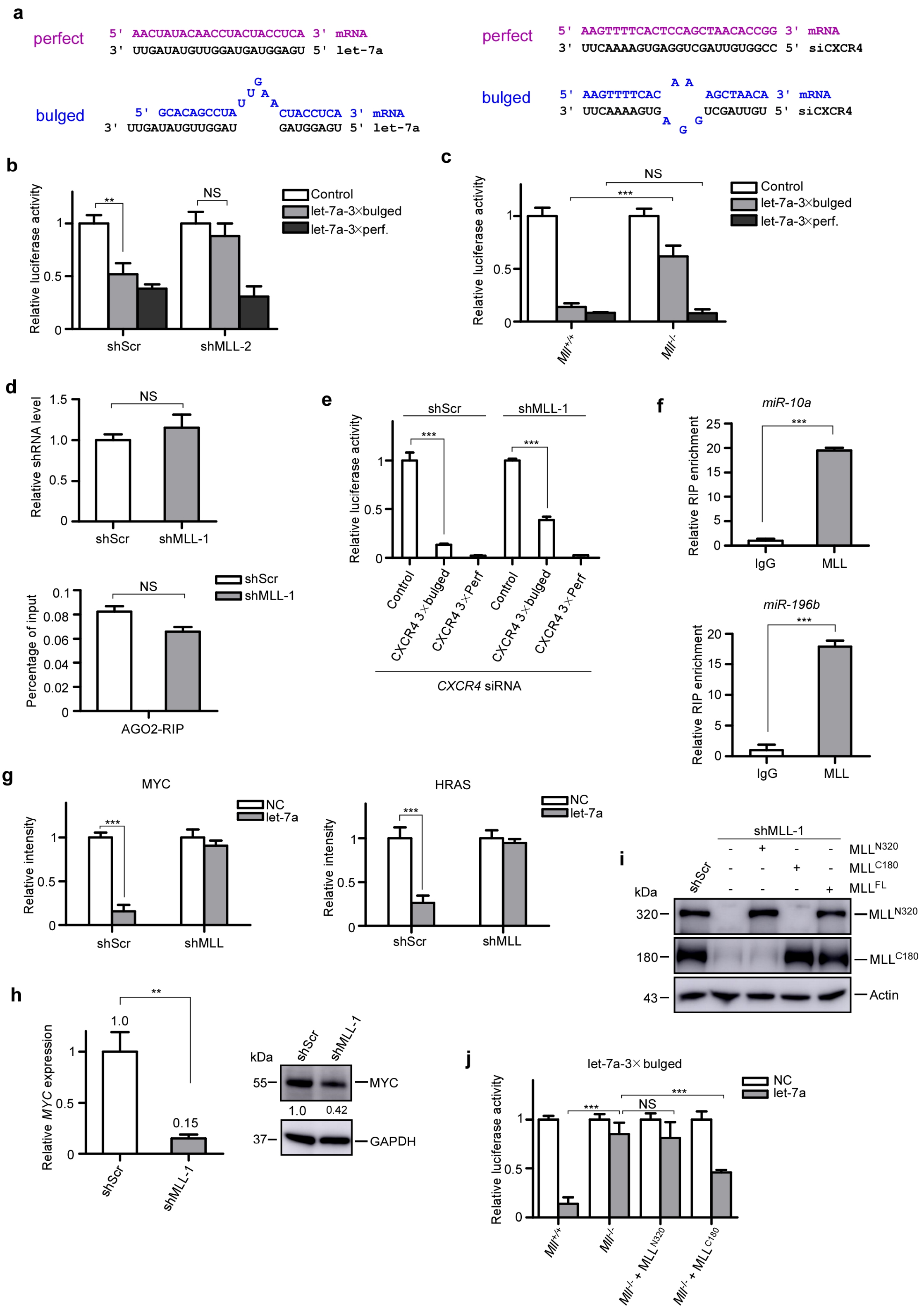

Supplementary Fig. S3 MLL is required for miRNA-mediated translational repression. a Sequences of perfect and bugled *let-7a* and *CXCR4* miRNA reporters. **b-c** Dual luciferase reporter assays were performed in 293T-shMLL cells (**b**) and *Mll* knockout (*Mll*$^{-/-}$) MEF cells (**c**) as described in Fig. 1l. The ratio

of luciferase activity was measured and normalized to the value of the cells transfected with the control reporter in each cell line. **d** (Upper panel): Relative expression levels of shRNA control and the MLL-targeting shRNA in 293T-shScr and 293T-shMLL cells were measured by qRT-PCR using specific primers. shRNA expression was normalized to *U6*. (Lower panel): shRNAs bound to AGO2 were quantified by RIP assays. **e** *CXCR4* dual luciferase reporters that contain 3 imperfect bulge target sites or 3 perfect target sites for siCXCR4 were used to evaluate the role for MLL in the miRNA-mediated gene silencing. The ratio of luciferase activity was normalized to the value of the cells co-transfected with the control reporter and siCXCR4 in each cell line. **f** Extracts of 293T cells were subjected to anti-MLL RIP analysis. Pull-downed RNAs were analyzed by qRT-PCR using specific primers for *hsa-miR-10a-5p* and *hsa-miR-196b-5p*. **g** Fold changes of MYC and HRAS proteins were indicated. Values were normalized to the 293T-shScr cells transfected with Agomir-negative control (NC). **h** MYC mRNA and protein levels in 293T cells were analyzed by qRT-PCR and western blot upon *MLL* depletion. *MYC* mRNA level was quantified relative to shScr. *GAPDH* was used as control in qRT-PCR experiments. Relative changes were indicated. **i** Transfections of *MLL* knockdown 293T cells with shRNA-resistant MLL^{N320}, MLL^{C180}, or full-length *MLL* (MLL^{FL}) were confirmed by western blot assays. Antibodies were used as indicated. **j** *Mll* knockout ($Mll^{-/-}$) MEF cells were transfected with MLL^{C180} or MLL^{N320} to determine which of these subunits could relief the dysregulated *let-7a* mediated gene silencing due to *Mll* knockout. Experiments were performed as described in Fig. 1k. The ratio of luciferase activity was measured and normalized to the value of the cells transfected with the NC. * for P<0.05, ** for P<0.01, *** for P<0.001. NS, no significant difference. Data represent mean and s.e.m of three independent experiments.

Supplementary Table S1. Proteins identified in MLL immunoprecipitation using mass spectrometry.

Supplementary Table S2. RIP-seq results using anti-MLL antibody for small RNA.

Supplementary Table S3. RIP-seq results using anti-MLL antibody for mRNA.
